# The complete chloroplast genome sequence of the medicinal plant *Persicaria filiformis*

**DOI:** 10.1080/23802359.2021.1950064

**Published:** 2021-07-09

**Authors:** Kun Zhang, Zhihui Gao, Zhiping Han, Xiaofei Shan

**Affiliations:** College of Life Sciences, Shanxi Datong University, Datong, China

**Keywords:** *Persicaria filiformis*, Polygonaceae, chloroplast genome, illumina sequencing

## Abstract

*Persicaria filiformis*, known as a traditional Chinese herbal medicine, is a perennial plant of Polygonaceae wildly distributed in China. The complete chloroplast (cp) genome of *P. filiformis* was assembled and analyzed in this study. The length of the circular genome is 159,741 bp, with a rich GC content of 41.3%. The cp genome structure consists of a large single-copy region (LSC 84,432 bp), a small single-copy region (SSC 13,073 bp) and a pair of inverted repeat regions (IR 31,118 bp). The complete genome encodes 130 genes, including 85 protein-coding genes, 37 tRNA genes and 8 rRNA genes. Phylogenetic analysis indicated that *P. filiformis* is most related to *P. japonica.*

*Persicaria filiformis* (Thunb.) Nakai is a specie of genus *Persicaria* in the plant family Polygonaceae represented by 50 genera and 1150 species around the world (Wang et al. 2015). The genus *Persicaria* includes three species and distributes in the East Asia and North America. Only one species and two varieties are originated in China (Ma et al. 2017). *Persicaria filiformis* is endemic to China, and scattered in the mountain forest margin in Guizhou, Shandong, Henan, Shanxi, Sichuan and Guangxi. In Flora of China, this species has been recognized as an *Antenoron filiforme* (Thunb.) Rob. et Vaut., which has two variants, namely *Antenoron filiforme* (Thunb.) Rob. et Vaut. var. neofiliforme (Nakai) and *Antenoron filiforme* (Thunb.) Rob. et Vaut. var. kachinum (Nieuw.), respectively. As a Chinese herbal medicine, *P. filiformis* is widely used in anti-inflammatory, analgesic and anticoagulant treatments for its significant pharmaceutical value. At present, relatively few reports have been made on the study of *P. filiformis*. The reported researches on *P. filiformis* mainly focused on phytochemical constituents (Zhao et al. 2011), pharmacological action (Huang et al. 2004; Ma et al. 2017) and phylogenetic origin (Xu et al. 2011). In recent years, the complete chloroplast (cp) genomes have been sequenced in diverse species, and comparative analysis with different close species cp genomes has provided an efficient means for revealing the genetic characteristics and evolutionary relationship of valuable species (Cai et al. 2020). The cp genome plays a more crucial role in the study of cp molecular markers development, species identification and population dynamics (Shaw et al. 2014). In the present study, we established and characterized the complete *P. filiformis* cp genome to provide valuable information for further genetic and phylogenetical studies of this plant.

The fresh leaves of *P. filiformis* were collected from Hengshan mountain in Datong, Shanxi, China (39°42′N, 113°41′E), and the voucher specimen (Accession number: HSEW202103) was deposited in Shanxi Datong University (http://www.sxdtdx.edu.cn/, Kun Zhang, 876828320@qq.com). The sample’s total genomic DNA was extracted from about 100 mg fresh leaves using a modified CTAB method (Murray and Thompson 1980). The libraries with an average length of 350 bp were constructed using the NexteraXT DNA Library Preparation Kit (Illumina, San Diego, CA), and then sequenced on Illumina Novaseq 6000 platform. Raw sequence reads were edited using NGS QC Tool kit (Patel and Jain 2012). In total, 3.7 Gb clean data was *de novo* assembled by SPAdes v.3.11.0 software (Bankevich et al. 2012) using the cp genome of *Persicaria chinensis* (L.) H. Gross (Accession number: MW627221) as the reference. Finally, the assembled complete cp genome was annotated via PGA (Qu et al. 2019). The annotated sequence was submitted to GenBank and data were openly available at (https://www.ncbi.nlm.nih.gov/nuccore/MW915468.1/) under the accession MW915468. The associated SRA number is SRR14160983.

The cp genome sequence of *P. filiformis* is 159,741 bp in length, with a quadripartite structure. Total GC content of this genome is 41.3%. The complete cp genome of *P. filiformis*, containing a large single-copy (LSC) region of 84,432 bp, a small single-copy (SSC) region of 13,073 bp and two inverted repeat (IR) regions of 31,118 bp. The cp genome encodes 130 genes, including 85 protein-coding genes, 37 tRNA genes and 8 rRNA genes. To further determine the phylogenetic position of *P. filiformis*, the complete cp genomes of other 19 species in Polygonaceae family and two species of Asphodelaceae as outgroup from GenBank were aligned using MAFFT (Katoh and Standley 2013, available at https://mafft.cbrc.jp/alignment/server/). Subsequently, a maximum-likelihood phylogenetic tree was established by IQTREE v1.6 (Jana et al. 2016) with 1000 bootstrap replicates. The phylogenetic analysis indicated that *P. filiformis* was closed to *Persicaria japonica* (Meisn.) H. Gross ex Nakai (Figure 1). The information derived from this work may help facilitate the identification and utilization of *P. filiformis*, and further elucidate the evolution of this medicinal plant.

**Figure 1.**
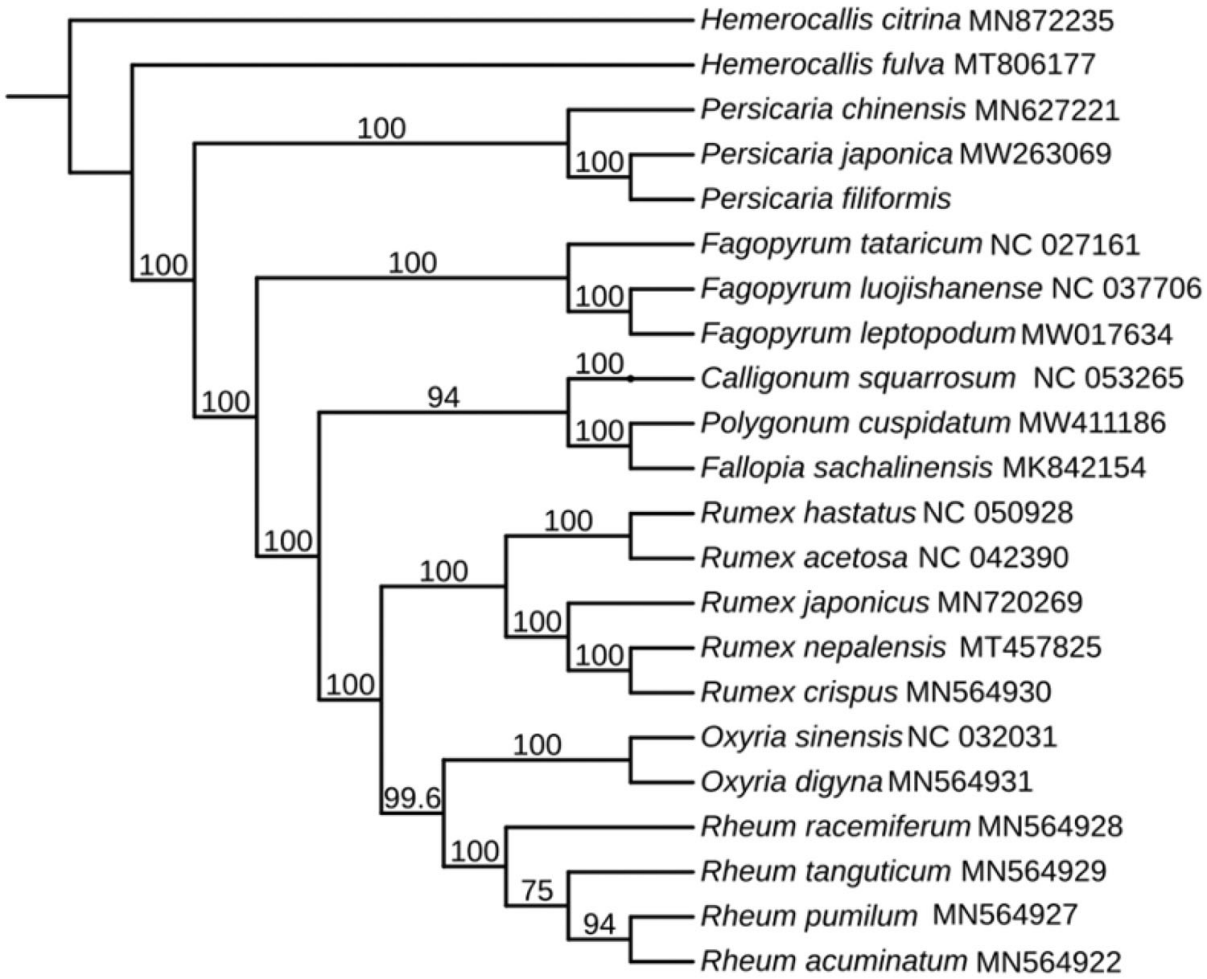
Maximum-likelihood phylogenetic tree for *P. filiformis* based on 20 complete cp genome in Polygonaceae, with *Hemerocallis citrina* Baroni and *Hemerocallis fulva* (L.) L. as outgroup. The bootstrap values are located on each node and the Genbank accession numbers are shown beside the Latin name of the species.

## Data Availability

The assembled complete cp genome sequence of *P. filiformis* has been submitted to GenBank of NCBI and are openly available under the accession number: MW915468 (https://www.ncbi.nlm.nih.gov/nuccore/MW915468.1/). The associated BioProject, SRA and Bio-Sample numbers are PRJNA720258, SRR14160983 and SAMN18644362 respectively.
